# Medical Qualifications

**Published:** 1903-09-05

**Authors:** 


### Medical Qualifications.

The first thing a medical student has to do is to register himself as such, because the five years of study which are necessary in order to obtain a qualification are dated from the time of registration. For this purpose application must be made to the Registrar of the General Medical Council, 299 Oxford Street, London, W.

The student, however, cannot be registered until he has (1) passed a preliminary examination in arts which is recognised by the General Medical Council, and (2) has commenced medical study.

A complete list of the recognised preliminary examinations may be obtained on application to the Registrar of the General Medical Council; and it is essential in any case to discover at a sufficiently early period what preliminary examinations are recognised by the particular examining body that it is proposed to work under. For example, with few exceptions the London Matriculation is the only preliminary examination which will permit a candidate to proceed t› the higher examinations in order to become a graduate of the University of London.

Wherever he proposes to study and whatever qualification he intends to get, the courses of study which a medical student has to undergo are really very much the same; and may be divided, generally speaking, into three groups, although of course the details of the courses will vary somewhat with the different examining bodies.

The first course consists of chemistry, physics, and biology; the second of anatomy and physiology; and the third of surgery and medicine and their various branches.

The following is a list of the examining bodies, and the degrees and diplomas granted by them which admit the graduate or diplomate to enter his name in the register of qualified medical men :—

ENGLAND.

| Examining Body. | Qualification. |
|---|---|
| University of Oxford ... ... | M.B., B Ch. (This degree is only obtainable after graduating in arts.) |
| University of Cambridge ... | M.B., B.C. |
| University of London ... ... | M B. |
| University of Durham ... ... | M.B. |
| Victoria University (Manchester and Leeds) ... ... ... | M.B., Ch.B. |
| University of Liverpool... ... | M.B., Ch.B. |
| University of Birmingham ... | M.B , B.Ch. |
| Conjoint Examining Board in England ... ... ... | M.R.C.S., L.R.C.P. |
| Society of Apothecaries of London... ... ... ... | L.S.A. |

SCOTLAND.

| | |
|---|---|
| University of Edinburgh ... | M.B., Ch.B. |
| University of Glasgow ... ... | M.B., Ch.B. |
| University of Aberdeen... ... | M.B., Ch.B. |
| University of St. Andrews ... | M.B., Ch.B. |
| Conjoint Examining Board in Scotland ... ... ... | L.R.C.S.Edin., L R.C.P.-Edin., & L.F.P.S.Glasg. |

IRELAND.

| | |
|---|---|
| University of Dublin ... ... | M.B., B.Ch., B.A.O. (These degrees are only conferred on graduates in arts ) |
| Royal University of Ireland ... | M.B., B.Ch. |
| Conjoint Examining Board in Ireland... ... ... ... | L.R.C.P.I., L.R C.S.I. |
| Apothecaries' Hall of Ireland ... | L.A.H.Dub. |

Many of these examining bodies lay down certain restrictions as to residence, and it is therefore important that an intending candidate should learn what these restrictions are by applying to the particular examining body from which he wishes to obtain his qualification.

